# GABA_A_ signaling, focal epileptiform synchronization and epileptogenesis

**DOI:** 10.3389/fncir.2022.984802

**Published:** 2022-10-05

**Authors:** Massimo Avoli, Marco de Curtis, Maxime Lévesque, Laura Librizzi, Laura Uva, Siyan Wang

**Affiliations:** ^1^Montreal Neurological Institute-Hospital, Montreal, QC, Canada; ^2^Departments of Neurology and Neurosurgery, Montreal, QC, Canada; ^3^Department of Physiology, McGill University, Montreal, QC, Canada; ^4^Epilepsy Unit, Fondazione Istituto di Ricovero e Cura a Carattere Scientifico (IRCCS), Istituto Neurologico Carlo Besta, Milan, Italy

**Keywords:** epileptiform synchronization, excitatory transmission, GABA_A_ receptor, inhibitory transmission, interictal spikes, mesial temporal lobe epilepsy, seizures

## Abstract

Under physiological conditions, neuronal network synchronization leads to different oscillatory EEG patterns that are associated with specific behavioral and cognitive functions. Excessive synchronization can, however, lead to focal or generalized epileptiform activities. It is indeed well established that in both epileptic patients and animal models, focal epileptiform EEG patterns are characterized by interictal and ictal (seizure) discharges. Over the last three decades, employing *in vitro* and *in vivo* recording techniques, several experimental studies have firmly identified a paradoxical role of GABA_A_ signaling in generating interictal discharges, and in initiating—and perhaps sustaining—focal seizures. Here, we will review these experiments and we will extend our appraisal to evidence suggesting that GABA_A_ signaling may also contribute to epileptogenesis, i.e., the development of plastic changes in brain excitability that leads to the chronic epileptic condition. Overall, we anticipate that this information should provide the rationale for developing new specific pharmacological treatments for patients presenting with focal epileptic disorders such as mesial temporal lobe epilepsy (MTLE).

## Background

Neuronal synchronization reflects the integrated activity occurring over time among neuronal networks that are located in the brain (Niedermeyer and da Silva, 2005). Under physiological conditions, neuronal synchronization results in different EEG oscillations that are associated with specific behavioral states, which include cognitive functions and sleep (Steriade et al., 1990; Buzsáki, 2015). However, neuronal synchronization can become abnormally excessive thus leading to focal (Jefferys et al., 2012; Avoli et al., 2016) and/or generalized epileptic discharges (Timofeev and Steriade, 2004; Crunelli et al., 2012). In this review, we will address the cellular and pharmacological mechanisms that cause the generation of epileptiform discharges in *in vivo* and *in vitro* animal models of focal epilepsy as well as in epileptic patients who were investigated with invasive electrophysiological recordings (including single unit activity) before undergoing brain surgery. These studies were performed in limbic brain structures—including the hippocampus, the rhinal cortices and the amygdala—since these areas are known to play a role in mesial temporal lobe epilepsy (MTLE) (Gloor, 1997; Engel et al., 2012).

**FIGURE 1:**
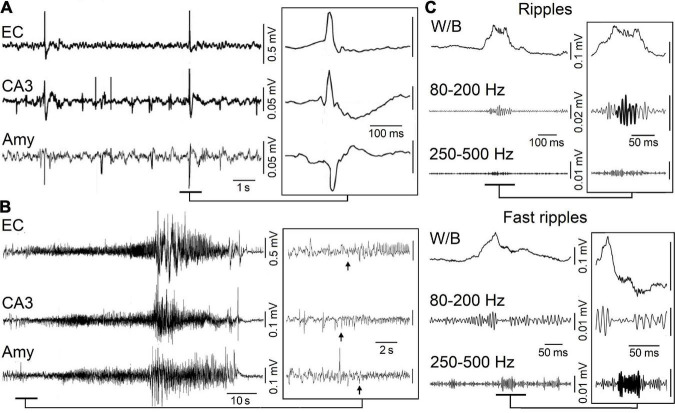
**(A)** Spontaneous interictal discharges recorded from the entorhinal cortex (EC), the hippocampal CA3 region and the amygdala (Amy) in a pilocarpine-treated epileptic rat. Note that only two interictal spikes are present in all regions. **(B)** Spontaneous ictal (seizure) discharge recorded in a pilocarpine-treated epileptic animal from the same areas as in **(A)**; low-voltage fast activity (arrows) marks the onset of this seizure. **(C)** High-frequency oscillations (HFOs, 80–500 Hz) recorded in association with interictal spikes in a pilocarpine-treated epileptic animal. Interictal spikes are visible on the wideband signal (W/B) whereas high-frequency activity is detectable only after filtering the signals between 80 and 200 Hz (Ripples) and between 250 and 500 Hz (Fast ripples).

Interictal discharges or spikes (i.e., short-lasting events with duration less than 1 s and unaccompanied by any detectable clinical symptom) (Figure 1A) as well as ictal discharges (i.e., periods of abnormal, hypersynchronous activity lasting up to several minutes and thus disrupting normal brain function) (Figure 1B) are recorded in the EEG obtained from animals or patients presenting with a focal epileptic condition such as MTLE (Gloor, 1997; de Curtis and Avanzini, 2001; Jefferys et al., 2012; Avoli et al., 2016). More recently, it has been shown that focal epileptiform activity is accompanied by the occurrence of high frequency oscillations (HFOs) in the EEG

(field potential) recordings (Figure 1C); HFOs are not visible in standard EEG recordings but can be extracted by amplifying the appropriately filtered signals. Based on their frequency content, they have been categorized in two groups: (i) ripples, which include oscillatory events between 80 and 200 Hz and (ii) fast ripples, i.e., oscillatory events occurring between 250 and 500 Hz (Bragin et al., 1999a,b; Staba et al., 2004; Jirsch et al., 2006; Urrestarazu et al., 2006; Foffani et al., 2007; Ibarz et al., 2010; Lévesque et al., 2011, 2012; Zijlmans et al., 2011). It has been proposed that ripples may represent, mainly, summated IPSPs while fast ripples should mirror synchronized action potential firing generated by principal (glutamatergic) cells (Jefferys et al., 2012; Jiruska et al., 2017), although fast-spiking GABAergic interneurons could also contribute to the generation of fast ripples (Cepeda et al., 2020). To note how interictal and ictal discharges along with HFOs share some common synchronizing mechanisms.

The topic of our review is the surprisingly active role played by GABA_A_ receptor signaling, in focal epileptiform synchronization. GABA_A_ receptors, once activated, open ionotropic anionic channels that are permeable to Cl^–^ and HCO_3_^–^ (Kaila, 1994). Early clinical evidence indicated that interfering with GABA synthesis leads to convulsions (Coursin, 1954). In addition, experimental studies, which were mainly published in the 1980s, revealed that: (i) several convulsive drugs are GABA_A_ receptor antagonists (Dingledine and Gjerstad, 1980; Schwartzkroin and Prince, 1980; Hablitz, 1984); (ii) inhibition is markedly reduced at the onset of electrographic hippocampal and neocortical seizures (Ben-Ari et al., 1979; Kostopoulos et al., 1983); (iii) functional disconnection of interneurons from excitatory inputs causes a decrease in inhibition in epileptic brains (Sloviter, 1987); (iv) inhibition in human MTLE may be reduced due to deficits in GABA transporter functions or alterations in GABA_A_ receptor subunit composition (McDonald et al., 1991; Johnson et al., 1992; Olsen et al., 1992; Williamson et al., 1995). Therefore, in the early 1990s, weakening of inhibition was considered by the majority of epilepsy researchers as the main mechanism leading to focal interictal and ictal discharges and thus to epileptic disorders. This view has been, however, challenged by several successive studies that will be summarized here. To note, however, that we will limit the focus of our review to experimental studies involving electrophysiology methods as it is not meant to cover studies involving other investigative approaches.

## GABA_A_ signaling and epileptiform synchronization

**FIGURE 2:**
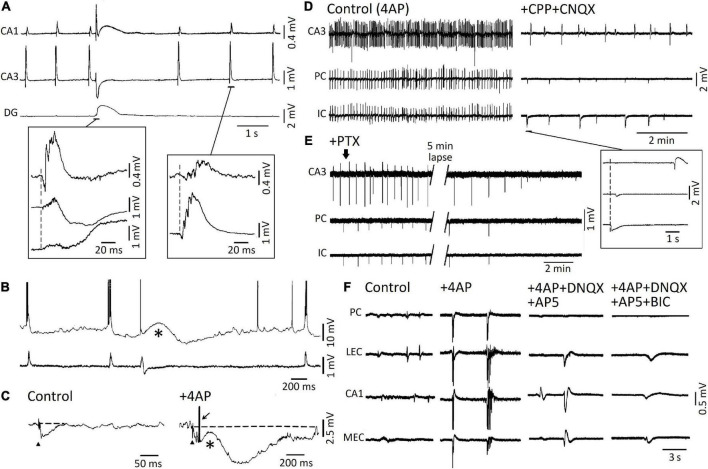
**(A)** Simultaneous field recordings obtained from CAI, CA3, and dentate gyrus in an adult rat hippocampal slice during 4AP application. Note that two types of spontaneous interictal spikes are spontaneously generated. The first type is long-lasting and less frequent, it is recorded in all three regions, and it is characterized by a late, “slow” wave; the second type is recorded in CA3 and CA1 only, it is characterized by a short burst of population spikes, and it occurs at a “fast” rate. **(B)** Simultaneous intracellular (top trace) and field potential (bottom trace) recordings obtained from the CA3 subfield during 4AP application. Note that intracellular bursts of action potentials correlated with the “fast” interictal field events, whereas a long-lasting depolarization corresponded to the “slow” interictal field event. **(C)** Responses to Schaffer collateral electrical stimulation (triangle) recorded intracellularly from a CA3 pyramidal cell under control conditions and in the presence of 4AP. Note that in control the presumptive recurrent hyperpolarizing IPSP lasts approx. 60 ms while, in the presence of 4AP, the same stimulation induces an initial hyperpolarizing IPSP followed by a slow depolarization (asterisk) and a long-lasting (almost 1 s long) hyperpolarization; the arrow indicates a fractionated, presumably ectopic, action potential that arises from the peak of the early hyperpolarizing IPSP. **(D)** Simultaneous field potential recordings obtained in an extended brain slice from the hippocampal CA3 area, the perirhinal cortex and the insular cortex under control (4AP) conditions and during application of NMDA and non-NMDA ionotropic glutamatergic receptor antagonists (+ CPP + CNQX). Note in the inset that during ionotropic glutamatergic receptor antagonism, “slow” field potentials continue to occur independently in CA3 from those seen *quasi* synchronously in PC and IC. **(E)** Under experimental conditions similar to those described for **(D)** (+ CPP + CNQX), addition of the GABA_A_ receptor antagonist picrotoxin abolishes the presumptive, “slow” GABAergic field potentials in all areas of the brain slice. **(F)** Epileptiform activity induced by 4AP arterial application in the piriform cortex (PC), lateral entorhinal cortex (l-EC), hippocampal CA1 subfield, and medial entorhinal cortex (m-EC) of the isolated brain preparation is greatly reduced by ionotropic glutamatergic receptor antagonists (+ DNQX + AP5). The residual field potential events are then abolished by further administration of the GABA_A_ receptor antagonist bicuculline (BIC). **(A,B)** Are modified from Perreault and Avoli (1992); **(C)** is modified from Perreault and Avoli (1991); **(D,E)** are modified from Sudbury and Avoli (2007); **(F)** is modified from Uva et al. (2009).

Voskuyl and Albus (1985) were the first investigators to report that a pharmacological procedure that does not decrease GABA_A_ receptor function—i.e., bath application of the K^+^blocker 4-aminopyridine (4AP)—can induce epileptiform activity in isolated rat hippocampal slices. By employing field potential recordings, they identified the spontaneous occurrence of two types of interictal spikes, with distinct shapes and rates of occurrence (Voskuyl and Albus, 1985). These two types of interictal patterns were confirmed to occur in successive studies in which field and intracellular potentials were simultaneously recorded from hippocampal slices (Perreault and Avoli, 1991, 1992). As shown in Figure 2A, field potential recordings obtained during 4AP application revealed: (i) “slow” interictal spikes occurring simultaneously in CA1, CA3, and dentate gyrus (DG), and (ii) “fast” interictal spikes that originate in CA3 and spread to CA1. Moreover, intracellular recordings from CA3 pyramidal cells demonstrated that “slow” interictal spikes were mirrored by slow depolarizations (which were abolished by GABA_A_ receptor antagonists), while “fast” interictal spikes were associated to intracellular bursts of action potentials riding on depolarizations that were caused by ionotropic glutamatergic currents (Figure 2B; Perreault and Avoli, 1991, 1992). It was also confirmed in these experiments (*cf.*, Buckle and Haas, 1982; Rutecki et al., 1987) that the postsynaptic responses caused by the activation of both GABA_A_ and, presumably, GABA_*B*_ receptors were not only preserved but greatly increased in amplitude and duration by 4AP (Figure 2C; Perreault and Avoli, 1991); to note as this complex, augmented response was characterized by a pronounced depolarizing component (asterisk in Figure 2C) that may be contributed by HCO_3_^–^—an anion that goes through the open GABA_A_ receptor and has an equilibrium potential more positive than Cl^–^ (Grover et al., 1993; Kaila, 1994)—as well as by the transient increase in extracellular [K^+^] caused by GABA_A_ receptor postsynaptic activation (*cf.* Kaila et al., 1997). Presumptive ectopic, fractionated action potentials (arrow in Figure 2C) could consistently be recorded during these “slow” stimulus-induced or spontaneous events (Avoli et al., 1998), and this evidence has been confirmed in neocortical interneurons as well (Keros and Hablitz, 2005).

The two types of 4AP-induced interictal spikes were later recorded in extended brain slices—which included the hippocampus proper and other limbic or para-limbic areas such as the entorhinal/perirhinal cortices, the amygdala and the insular cortex (Figure 2D; Avoli et al., 1996a,b; Sudbury and Avoli, 2007)—as well as in the *in vitro* guinea pig isolated brain (Figure 2F; Uva et al., 2009). These studies (see also Morris et al., 1996; Lamsa and Kaila, 1997) have demonstrated that “fast” interictal spikes are abolished by ionotropic glutamatergic antagonists, a pharmacological procedure that does not appear to influence the recurrence of “slow” interictal spikes (Figures 2D,F), which are, however, eliminated by application of the GABA_A_; receptor antagonists picrotoxin (Figure 2E) or bicuculline (Figure 2F) as well as by activating μ-opioid receptors (Avoli et al., 1996a,b); this pharmacological procedure abolishes the presynaptic release of GABA (Capogna et al., 1993).

As shown in Figures 2D,F, slow, glutamatergic independent, interictal events continued to propagate through the extended brain slice and in the guinea pig isolated brain. As further discussed below, such propagation may depend on the increases in extracellular [K^+^] that accompany the slow interictal spikes induced by 4AP. To note as two types of interictal spikes have been identified in *in vivo* EEG recordings obtained from epileptic animals, and have been thereafter termed “type 1” and “type 2” (Bortel et al., 2010; Chauvière et al., 2012; Salami et al., 2014; Lévesque et al., 2021b). It should also be emphasized that preservation of inhibition is present in several *in vitro* models of epileptiform interictal synchronization such as those induced by application of Mg^2+^ free-medium (Mody et al., 1987; Tancredi et al., 1990), high K^+^ medium (Rutecki et al., 1985) or tetraethylammonium (Rutecki et al., 1990).

**FIGURE 3:**
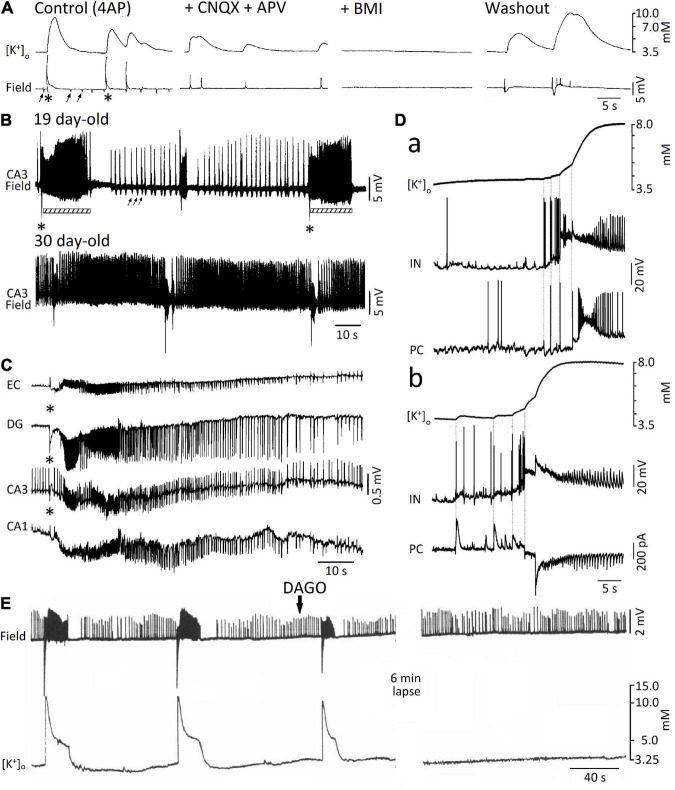
**(A)** Simultaneous extracellular [K^+^] and field potential recordings obtained from the CA1 subfield of an adult rat hippocampal slice during 4AP (Control) and successive application of ionotropic glutamatergic receptor antagonists (+ CNQX + CPP), GABA_A_ receptor antagonist (BMI), and washout (i.e., return to 4AP application for over 3 h); note in control the occurrence of both “slow” (asterisks) and “fast” (arrows) interictal spikes as well as that only the “slow” spikes are associated with sizable increases in extracellular [K^+^]. Note also that these “slow” spikes continue to occur during CNQX + CPP but are abolished by (BMI). **(B)** Field potential recordings from the CA3 *stratum radiatum* of two hippocampal slices obtained from 19 and 30 day-old rats during application of 4AP; note that at day 19, “fast” (arrows) and “slow” (asterisks) interictal spikes occur along with ictal discharges (bars) that are shortly preceded by a low interictal; ictal discharges are, however, not recorded in the experiment performed at postnatal day 30. **(C)** Simultaneous field potential recordings obtained from the entorhinal cortex (EC) and from the hippocampal the dentate gyrus (DG) and CA1 and CA3 subfields during application of 4AP in an extended brain slice; note that the “slow” (asterisk), along with the subsequent ictal discharge, are recorded from all areas while the “fast” interictal spikes are clearly detected in CA3 only. **(D)** Simultaneous extracellular [K^+^] and intracellular recordings from fast-spiking interneurons (IN) and principal cells (PC) in the medial entorhinal cortex at the onset of two ictal discharges occurring during 4AP application. Current-clamp recordings from both IN and PC are shown in **(a)**, while current -clamp and voltage-clamp recordings from and IN and a PC, respectively, are illustrated in **(b)**. Note that in both examples the IN fires action potentials earlier than the PC and that these firings correspond to time-locked elevations in extracellular [K^+^]; note also in **(b)** that interneuron firing is mirrored by outward currents in the PC. **(E)** Effects induced by the μ-opioid receptor agonist DAGO on the epileptiform activity recorded from the CA3 of a 15-day-old rat brain slice during 4AP application; note that the negative-going, “slow” spikes and the subsequent ictal activity along with the associated increases in extracellular [K^+^] are abolished by DAGO, and are replaced by continuous “fast” interictal events. **(A)** Is modified from Morris et al. (1996); **(B)** is modified from Avoli (1990); **(C)** is modified from Avoli et al. (1996a); **(D)** is modified from Librizzi et al. (2017); **(E)** is modified from Avoli et al. (1996b).

The likely role played by elevations in extracellular [K^+^] in the spread of the “slow,” mainly GABAergic, interictal spikes recorded during application of 4AP and ionotropic glutamatergic antagonists was originally proposed by Perreault and Avoli (1992). Shortly before, Barolet and Morris (1991) had discovered that GABA_A_ receptor activation, resulting from the application of exogenous GABA or the GABA_A_ receptor agonist THIP, led to increases in extracellular [K^+^] even when voltage-gated Na^+^ channels were blocked by tetrodotoxin, thus excluding any relevant contribution of action potential firing to such elevations in extracellular [K^+^]. As illustrated in Figure 3A, a few years later, Morris et al. (1996) reported that the “slow,” 4AP-induced spikes recorded from different regions of the isolated, adult rat hippocampal slice are mirrored by increases in extracellular [K^+^] that continue to occur in the presence of the ionotropic glutamate receptor antagonists 6- cyano-7-nitroquinoxalone-2,3-dione (CNQX) and DL-2- amino-5-phosphonovaleric acid (APV); however, these field events—along with their associated increases in extracellular [K^+^]—were reversibly blocked by the GABA_A_ receptor antagonist bicuculline methiodide (BMI). Similar data have been obtained in successive studies that were aimed at analyzing the elevations in extracellular [K^+^] associated to the “slow” interictal spikes induced by 4AP in slices of the rat hippocampus (Avoli et al., 1996b; Lamsa and Kaila, 1997), the rat or mouse entorhinal cortex (Avoli et al., 1996a; Librizzi et al., 2017) and the human neocortex (Louvel et al., 2001; D’Antuono et al., 2004). Extracellular [K^+^] elevations associated to GABA_A_ receptor-mediated spikes were also shown to occur in the entorhinal cortex of the *in vitro* isolated whole guinea pig brain (Librizzi et al., 2017). Overall, these data indicate that slow interictal spikes induced by 4AP mainly result from synchronous firing of interneurons that causes massive release of GABA, subsequent activation of post-synaptic GABA_A_ receptors and thus sizeable increases in extracellular [K^+^] through the activation of the KCC2 cotransporter (Viitanen et al., 2010).

A turning point on the role played by GABA_A_ receptor signaling in epileptiform synchronization coincided with the discovery that the onset of ictal discharges recorded from juvenile (15–22 day-old) rat hippocampal slices during 4AP application, is shortly preceded, and thus presumably caused by a field event that resemble the “slow” GABAergic spike (asterisk in Figure 3B, 19 day-old field recording) (Avoli, 1990; Avoli et al., 1993, 1996b); to note how this interictal-ictal pattern disappeared with brain maturation to be replaced by a continuous pattern of “fast” and “slow” interictal spikes (Figure 3B, 30 day-old field recording) (*cf.* also Psarropoulou and Avoli, 1996). However, successive *in vitro* studies, which were performed in extended brain slices and in the isolated guinea pig brain revealed that ictal (seizure-like) discharges can occur in adult brain tissue during 4AP application as well as that they are initiated and presumably maintained by GABA_A_ receptor signaling (Figure 3C; Avoli et al., 1996a,^2004^; Sudbury and Avoli, 2007; Carriero et al., 2010; Uva et al., 2013, 2015; Librizzi et al., 2017). The role of GABA_A_ receptor signaling in the initiation of seizure-like activity has been confirmed by computational studies; Kurbatova et al. (2016) have indeed reported that before seizure onset, high frequency firing of GABAergic interneurons generates an increase of the depolarizing GABA_A_ onto pyramidal cells, which induces a massive drop of inhibition that may allow seizure initiation. Interestingly, it has been also shown that the fast activity that occurs at seizure onset and characterizes low-voltage fast onset seizures is associated with interneuron firing, while pyramidal cells remain silent. Employing a biophysically network model (González et al., 2018), have also reported, that seizure-like activity triggered by interneuron firing would not depend on depolarizing GABA_A_ signaling, but would instead rely on an increase of intracellular [Cl^–^], which is sufficient for KCC2 activation, the subsequent accumulation of extracellular [K^+^] and the development of epileptiform activity (González et al., 2018).

In line with the mechanism discussed above (i.e., that interneuron firing leading to GABA_A_ receptor activation does, in turn, cause sizeable elevations in extracellular [K^+^]), several studies have reported that the initial (sentinel) spikes preceding the ictal events induced by 4AP is associated with interneuron action potential firing along with a large increase in extracellular [K^+^] (Avoli et al., 1996a,b; Ziburkus et al., 2006; Lévesque et al., 2016; Librizzi et al., 2017). This aspect is further illustrated in Figure 3D. First, double patch-clamp recordings of an interneuron and a principal cell in mouse entorhinal cortex slices demonstrated that interneuron burst discharges coupled with IPSPs (Figure 3Da) or IPSCs (Figure 3Db) in principal neurons occur at the onset of 4AP-induced ictal activity; second, such “pre-ictal” patterns were associated with rises in extracellular [K^+^] that were closely related to interneuron firing and further enhanced by the ensuing recruitment of neuronal networks into the seizure activity. These results firmly support the view that elevations in extracellular [K^+^] are caused by interneuron firing, which consistently precedes the initiation of ictal events as well as that these extracellular [K^+^] elevations contribute to seizure precipitation. Interestingly, the emergence of seizure-like activity during extracellular [K^+^] perturbations has been demonstrated by a realistic computational model of cortical networks (Fröhlich et al., 2010).

It is well known that elevating extracellular [K^+^] induces neuronal hyperexcitability along with seizure activity (Zuckermann and Glaser, 1968). Successive studies have demonstrated that increased extracellular [K^+^] causes a positive shift of the membrane reversal of the GABA_A_ receptor-mediated currents thus weakening inhibition (Jensen et al., 1993); it has also been shown that neuronal network resonance, which leads to oscillatory patterns in the beta-gamma range, emerges during increased extracellular [K^+^] (Bartos et al., 2007). These data are therefore in line with the role played by GABA_A_ receptor activation in promoting epileptiform synchronization and thus seizure-like activity. To be emphasized as pharmacological procedures that interfere with GABA_A_ signaling (e.g., GABA_A_ receptor antagonists or μ-opioid receptor agonists) halt ictal discharges induced *in vitro* by 4AP and replace them with a pattern of recurring, short-lasting interictal spikes (Figure 3E; Avoli et al., 1996a,b, 2004; Sudbury and Avoli, 2007). In human epileptic tissue, blockade of GABA_A_ receptors also halts interictal discharges (Cohen et al., 2002; Blauwblomme et al., 2019) or modifies their spatial propagation (Sabolek et al., 2012). Suppression of interictal discharges can also be obtained with the application of the NKCC1 blocker bumetanide, in brain slices obtained from pediatric patients with focal cortical dysplasia (Blauwblomme et al., 2019) or in slices obtained from patients with temporal lobe epilepsy and hippocampal sclerosis (Huberfeld et al., 2007), therefore suggesting that the depolarizing responses to GABA in a subset of pyramidal cells during interictal spikes results from excessively high intracellular [Cl^–^]. A depolarizing action of GABA due to altered intracellular [Cl^–^] homeostasis has also been demonstrated in tissue obtained from pediatric patients with cortical dysplasia (Abdijadid et al., 2015).

**FIGURE 4:**
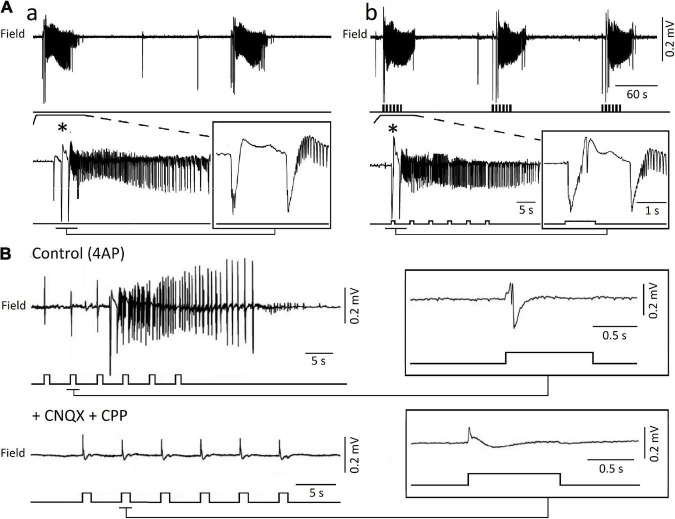
**(A)** Ictal discharges recorded extracellularly from the mouse entorhinal cortex during application of 4AP can occur spontaneously **(a)** or be triggered by optogenetic activation of parvalbumin interneurons **(b)**. One ictal event for each experimental condition is further expanded to show the onset patterns that are in both cases characterized by 1 or 2 negative-going interictal-like spikes. **(B)** Blockade of ionotropic glutamatergic receptors (+ CNQX + CPP) abolishes ictal discharges induced by the optogenetic activation of parvalbumin-positive interneurons in the presence of 4AP; however, under these experimental conditions optogenetic stimuli continue to evoke slow interictal spike. **(A,B)** Are modified from Shiri et al. (2016). The onset of the ictal discharge (*) is shown on an expanded time scale in the inset.

The paradoxical role played by GABA_A_ receptors in initiating 4AP-induced ictal (seizure-like) events (*cf.*, de Curtis and Avoli, 2016) has been confirmed by studies in which optogenetic activation of parvalbumin- or somatostatin-positive interneurons was found capable of triggering ictal events with electrographic features similar to those occurring spontaneously (Shiri et al., 2015, 2016; Yekhlef et al., 2015; Lado et al., 2022). As illustrated in Figure 4A, optogenetic activation of parvalbumin-positive interneurons in the entorhinal cortex (panel b) initiates local ictal discharges that are characterized by an onset that is superimposable to what recorded during spontaneous events (panel a); in fact; the onset of both spontaneous and optogenetic-induced ictal events is typified by one-two interictal-like spikes that lead to fast, beta-gamma oscillations, which characterize the initial component of the seizure activity; these electrographic characteristics represent the hallmark of low-voltage fast onset ictal discharges recorded in patients presenting with focal epileptic disorders (Perucca et al., 2014) and in animal models *in vivo* (Lévesque et al., 2012). Moreover, it was found in these experiments that optogenetic activation of parvalbumin-positive interneurons could evoke slow interictal spikes both during application of 4AP and after blockade of ionotropic excitatory transmission (Figure 4B; Shiri et al., 2016).

The surprisingly active role played by GABA_A_ signaling in initiating and, perhaps, sustaining seizure activity *in vitro* has been identified under different experimental conditions, including perfusion of low doses of bicuculline in the isolated guinea pig brain (Gnatkovsky et al., 2008), perfusion of brain slices with Mg^2+^ free medium (Köhling et al., 2000), or high frequency electrical stimuli (Velazquez and Carlen, 1999; Fujiwara-Tsukamoto et al., 2010). The contribution of GABA_A_ receptors to epileptiform synchronization is also supported by the ability of CA1 hippocampal networks *in vitro* to generate prolonged discharges following pharmacological blockade of both GABA_*B*_ and ionotropic glutamatergic receptors (Uusisaari et al., 2002). Evidence obtained from *in vivo* models of MTLE have also shown that increased activity of GABA releasing interneurons (which in turns silences principal neurons) coincides with the onset of focal seizures (Grasse et al., 2013; Fujita et al., 2014; Toyoda et al., 2015; Karunakaran et al., 2016). Last but not least, seizure onsets recorded from epileptic patients undergoing presurgical depth electrode investigations, is associated with increased interneuron firing and marked reduction of principal cell excitability (Truccolo et al., 2011; Schevon et al., 2012; Elahian et al., 2018).

## GABA_A_ signaling and epileptiform discharges *in vivo*

The kainic acid (KA) (Lévesque and Avoli, 2013) and the pilocarpine models of MTLE (Lévesque et al., 2021a) have been widely used to study how epileptic discharges are generated from mesial temporal lobe structures *in vivo*. Both models rely on the chemical induction of an initial brain insult (i.e., a *status epilepticus*, SE), that is followed a few days later by the development of a chronic epileptic condition. GABA_A_ signaling could play a role in ictogenesis in these animal models, since alterations in GABA_A_ receptor function and in GABA releasing interneurons have been reported (Friedman et al., 1994; Schwarzer et al., 1997; Tsunashima et al., 1997; Laurén et al., 2005; Fritsch et al., 2009; Drexel et al., 2013; Dubanet et al., 2021).

In the KA model, spontaneous seizures occurring in epileptic mice can be stopped, and the frequency of seizures with severe behavioral symptoms reduced, when optogenetic activation of ChR2-expressing PV-positive interneurons is performed in the hippocampus ipsilateral or contralateral to the hippocampus that was injected with KA (Krook-Magnuson et al., 2013). Similar findings were obtained by Chen et al. (2021), who used optogenetics in KA-treated epileptic animals to activate hippocampal PV-interneurons expressing ChRmine; this is a red-shifted opsin that exhibits high sensitivity to light stimulation (Marshel et al., 2019), thus making neurons expressing these opsins sensitive to transcranial optogenetic stimulation. The application of on-demand transcranial optogenetic stimulation to these ChRmine-expressing PV-positive interneurons during the chronic period induced a 51% decrease in seizure duration compared to sham treatment. Interestingly, optogenetic activation of PV-positive interneurons in the hippocampus of KA-treated animals also improves performance in cognitive tasks (Kim et al., 2020).

Such anti-ictogenic effect is not restricted to the hippocampus but it is also observed when optogenetic stimulation is applied to PV-expressing Purkinje cells of the cerebellum, a brain structure that is anatomically and functionally connected to the hippocampus (Watson et al., 2018) and that is known to modulate hippocampal function during cognitive tasks (Zeidler et al., 2020). Krook-Magnuson et al. (2014) found that optogenetic excitation or inhibition of PV-expressing Purkinje cells in the lateral or midline cerebellum of KA-treated animals during the chronic period shortens seizure duration. However, it remains unclear through which mechanisms cerebellar optogenetic stimulation controls hippocampal seizures, since both excitation and inhibition of cerebellar Purkinje cells could decrease seizure duration (Krook-Magnuson et al., 2014). Similar anti-ictogenic effects in the KA model resulting from the activation of GABAergic neuronal populations in remote regions were also reported recently by Hristova et al. (2021), who performed optogenetic stimulation of GABAergic populations in the medial septum, a region that sends GABAergic projections to hippocampal GABAergic interneurons (Unal et al., 2015).

**FIGURE 5:**
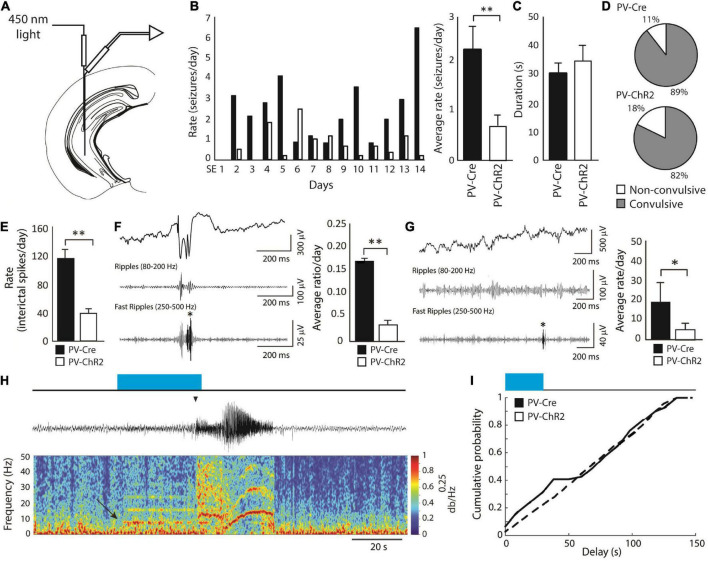
**(A)** Schematic diagram showing the location of the optic fiber and electrode in the CA3 region of the hippocampus. The tip of the optic fiber was glued less than 1 mm above the tip of the electrode. Optogenetic stimulation of PV-positive interneurons (8 Hz for 30 s every 2 min) was performed for 14 continuous days, starting 3 days after SE. **(B)** Average daily rates of spontaneous seizures in PV-ChR2 and PV-Cre animals. PV-ChR2 animals showed significantly less seizures compared to PV-Cre animals (^**^p < 0.005). **(C)** Bar graph showing the average duration of seizures in both groups. No significant differences were observed. **(D)** Proportion of non-convulsive and convulsive seizures in both groups. No significant differences were observed. **(E)** Bar graph showing rates of interictal spikes in both groups. PV-Cre animals showed significantly higher rates of interictal spikes compared to PV-ChR2 animals (^**^p < 0.001). **(F)** Bar graph showing the average ratio of interictal spikes with fast ripples on the total number of interictal spikes for each group. PV-Cre animals showed a higher ratio compared to the PV-ChR2 group (^**^p < 0.001). A representative example of an interictal spike with a fast ripple is shown on the right. **(G)** Bar graph showing the average rate of isolated fast ripples in both groups. PV-ChR2 animals showed significantly lower rates of isolated fast ripples compared to PV-Cre animals (*p < 0.01). **(H)** Example of a spontaneous seizure that was triggered by optogenetic stimulation of PV-positive interneurons (blue rectangle) in a PV-ChR2 animal. Note that oscillations around 8 Hz in the field (arrow) were triggered by light stimulation and that the seizure occurred approximately 25 s after (arrowhead). **(I)** Cumulative probability curves showing that PV-ChR2 animals are more likely to show seizures between 0 and 30 s after the onset of optogenetic stimulation compared to PV-Cre animals. Modified from Lévesque et al. (2019).

In the pilocarpine model, Lévesque et al. (2019) investigated whether continuous, unilateral, optogenetic stimulation of ChR2-expressing PV-positive interneurons in the CA3 subfield of the hippocampus (Figure 5A) could decrease seizure rates in pilocarpine-treated epileptic mice. These results have revealed that activation of PV-ChR2 interneurons at 8 Hz for 30 s every 2 min for 14 continuous days induce a decrease in rates of spontaneous seizures compared to what was observed in PV-Cre (opsin-negative) animals (Figure 5B). Seizure duration (Figure 5C) and proportion of convulsive seizures (Figure 5D) were not decreased by PV optogenetic stimulation; however, rates of interictal spikes (Figure 5E), of interictal spikes with fast ripples (Figure 5F) and of isolated fast ripples (Figure 5G)—which are considered as markers of epileptogenesis (Jefferys et al., 2012)–were significantly lower in the PV-ChR2 group compared to the PV-Cre group.

These findings are in line with the evidence obtained by Krook-Magnuson et al. (2013), who reported a decrease in seizure rates in the KA model by using closed-loop activation of PV-positive interneurons. However, Lévesque et al. (2019) also found that the “residual” seizures that continued to occur, could be triggered by optogenetic stimuli (Figures 5H,I). These data are in line with what was reported *in vitro* (Shiri et al., 2015, 2016; Yekhlef et al., 2015; Chang et al., 2018; Botterill et al., 2019).

## Concluding remarks

The studies reviewed here disclose an unexpected role played by GABA_A_ receptors in epileptiform synchronization including the generation of interictal and ictal (seizure) events. Such paradoxical role depends on the large increases in extracellular [K^+^] that are caused by KCC2 activation due to massive release of GABA consequent to synchronous firing of inhibitory interneurons (Di Cristo et al., 2018). We have also summarized recent findings suggesting that activation of inhibitory interneurons can exert unexpected effects on the processes associated to epileptogenesis. These results reveal a complex pattern of participating mechanisms. Thus, while synaptic excitation and voltage-gated Na^+^ channels remain the key components of synchronous epileptiform discharges, GABA_A_ receptors have emerged as surprising, paradoxical players in the generation of interictal spikes and in the initiation and maintenance of prolonged epileptiform phenomena (i.e., to ictogenesis).

The evidence that enhanced GABA_A_ receptor function supports epileptiform synchronization and thus focal seizure generation may explain the disappointingly limited clinical efficacy of some antiepileptic compounds that were “mechanistically” developed to potentiate GABA_A_ signaling during the 1980s and were introduced into clinical practice at the start of the 1990s. These compounds include γ-vinyl-GABA (which inhibits the breakdown of GABA by the enzyme GABA transaminase) (Rogawski and Löscher, 2004), tiagabine (which increases GABA levels by inhibiting GABA reuptake) (Brodie, 1995; Pollack et al., 2005), and progabide (Lloyd et al., 1983; Loiseau et al., 1983). It should also be emphasized that benzodiazepines, which can halt seizure activity and stop *status epilepticus* (Pang and Hirsch, 2005), increase GABA_A_ receptor function by acting on an allosteric “benzodiazepine site” that is located in most of the α subunit-containing GABA_A_ receptors (Costa et al., 1975; Choi et al., 1977; Olsen, 2015). However, and in line with a synchronizing action of GABA, benzodiazepines have been reported to precipitate seizures, when given intravenously in patients with Lennox-Gastaut syndrome (Perucca et al., 1998).

## Data availability statement

The original contributions presented in this study are included in the article/supplementary material, further inquiries can be directed to the corresponding author.

## Author contributions

MA wrote the early draft of this review. All authors contributed to the manuscript revision, read, and approved the submitted version.
